# Taguchi Grey relational analysis (GRA) based multi response optimization of flammability, comfort and mechanical properties in station suits

**DOI:** 10.1016/j.heliyon.2025.e42508

**Published:** 2025-02-07

**Authors:** Hafsa Jamshaid, Awais Ahmad Khan, Rajesh Kumar Mishra, Naseer Ahmad, Vijay Chandan, Viktor Kolář, Miroslav Müller

**Affiliations:** a School of Engineering and Technology, National Textile University, Sheikhupura Road, Faisalabad, 37610, Pakistan; b Department of Material Science and Manufacturing Technology, Faculty of Engineering, Czech University of Life Sciences Prague, Kamycka 129, Suchdol, 165 00, Prague, Czech Republic; c School of Sciences, National Textile University, Sheikhupura Road, Faisalabad, 37610, Pakistan

**Keywords:** Fire-resistant fabrics, Grey relational analysis (GRA), Analysis of variance, Station suits, Flammability, Thermo-physiological comfort

## Abstract

Fire fighters’ stations suits are expected to have adequate flame resistance for protection of the wearer. At the same time the comfort of wearing is essential to prevent fatigue and to maintain the efficiency during long working hours. Therefore, it is essential to balance these multi responses in a scientific manner. This study explores the multi-response optimization of fiber blend composition and knitted structures on the responses like flammability, comfort, and serviceability/mechanical properties, for the development of fire-resistant fabrics. Inherently fire-resistant (FR) fibers like meta-aramid Nomex, FR-Viscose, modacrylic (Protex) and carbon/Pyron fibers (Zoltek) were used in different blends to form yarns. Interlock derivative knitted structures like Cross Relief, Cross Miss and Vertical Tubular were developed. The results have been analyzed using Taguchi Grey relational analysis (GRA) for multi-response optimization. The analysis revealed that the sample with the blend of 70/30 % Protex/Nomex fiber and a Cross-relief structure ranked the best sample in terms of required performance. The percentage contribution of each factor was analyzed using ANOVA (α = 0.05) and visualized using a pie chart. While Factor B (Knitted Structure) demonstrated the highest variance contribution (42.3 %), it was not statistically significant based on the p-value and F-ratio from ANOVA results. The findings of this study are useful for designing flame-resistant uniforms/Station garment for firefighters without compromising wearing comfort so that the efficiency of the workers will not be compromised.

## Graphical abstract

**Figure:**
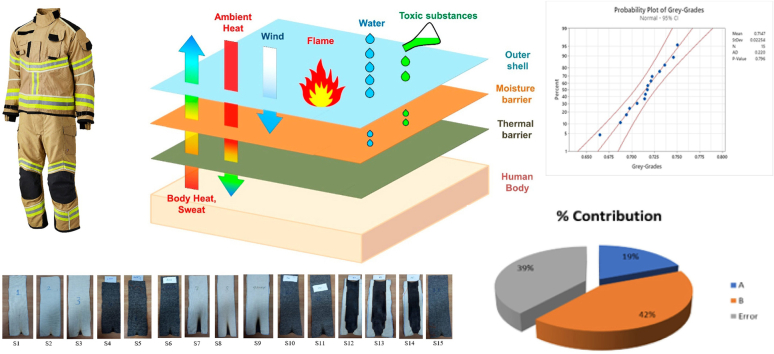

## 1 Introduction

Fire fighters’ clothing is expected to provide them with protection against flame and heat. They perform their duties under extremely dangerous conditions in which they are exposed to various types of accidents related to fire which include flames, metal splashes, smoke, explosions, noise, thermal stress and high temperatures. The station suits or the undergarments worn next to the skin under multilayer fire protective clothing should ensure preventing tissue damage upon exposure to the intense thermal conditions. Although fire protective clothing provides ultimate protection to the body, but while working under intense heat, it is very important to consider the microclimate developing between the body and the clothing next to the skin. To provide ultimate comfort, there is a need to maintain this microclimate by the various factors including air permeability, moisture management, thermal resistance etc. [1]. Due to intense activities, the heat generated by the body metabolism and the sweat should dissipate to the outside environment. Otherwise, it will also cause heat stroke. The station suits must have optimized fire resistance as well as the comfort properties. Use of performance certified station suits with better thermal comfort, ergonomic mobility and protection against fire & injury are the essential requirements of the overall turnout assembly. Researchers are investigating in different directions to develop high performance station wear to provide ultimate protection, comfort and other performance to the fire fighters in low risk as well as high risk tasks [2].

Apart from fabric structure, the fiber material used in station suits plays a very crucial role since they are in a direct contact with the skin. The material used should be capable to effectively drain away the sweat. Another important consideration is to use a material which prevents static charge development such as cotton blended with other high-performance fibers [3]. It has been recommended based on the research work that meta aramid fibers should be used in the development of firefighters underwear fabrics to provide ultimate protection [4]. Researchers have developed different knitted fabrics using superabsorbent Polyacrylate for station suits of fire fighters and evaluated their performance. The properties were compared with woven fabrics, and it was found that knitted fabrics show better performance [5]. Under wear fabrics were produced using 70 % wool & 30 % Modacrylic fibers with knitted fabrics and the performance of these suits were evaluated using thermal manikin. It was also suggested that without using such underwear, the fire fighters have higher risk of getting more injuries. Therefore, to avoid these issues the usage of station suits composed of blended material is very beneficial [6]. It was found that fiber composition and fabric structural characteristics play a very important role in providing comfort and protection. The melting and dripping behavior of polymeric fibers is very important consideration because it increases the risk of injury [7]. Knitted station suits worn next to the skin were developed using different fiber blends of cotton and modacrylic fibers. Various properties including fire retardancy, mechanical and comfort properties of the single jersey, rib, interlock and fleece fabrics composed of different blend ratios of modacrylic fibers were evaluated. It was recommended that these samples can be used under different high temperature conditions [8]. Phase change materials (PCM) were also incorporated by researchers to regulate the temperature, prevent heat stroke and provide ultimate comfort to the wearer [9]. The core temperature of fire fighters’ skin was studied and mathematical models were developed which assist in the estimation in the increment of core skin temperature during their activity in firefighting [10]. Apart from protective performance, comfort properties are very essential aspect of good fire protective fabrics. So, to develop fire protective clothing with better fire resistant and comfort properties there is need to optimize various parameters in the course of production of fire-resistant fabrics. Statistical techniques have the prominent potential to be used in various fields to optimize the process parameters. Similarly, for evaluating, optimizing and predicting the performance of various textile fabrics and structures, statistical tools are very useful. Various statistical tools like analysis of variance (ANOVA) were used to statistically analyze the effect of various effecting factors. The conventional fire protective clothing and advanced suits comprising PCM were statistically compared using correlation analysis. It was manifested that firefighting suits containing PCM are more feasible than its traditional counter parts due to improved overall performance [11]. Response surface methodology was used to statistically evaluate the effect of fabric horizontal and vertical orientation on the performance of fire protective suits. Moreover, different parameters were optimized using response surface methodology [12].

To the best of authors knowledge, no work has been reported on multiple-response optimization using Taguchi Grey relational analysis (GRA) for FR station suits/engineered FR material for multiple responses. Therefore, the aim of this study is to present an effective method for optimizing the fire resistant (FR), comfort, mechanical and dimensional properties of knitted structures and fiber blends. Analysis of variance (ANOVA) was also used in conjunction with the Taguchi-grey relational analysis to detect the significant factors affecting the performance characteristics/responses. The contribution percentage of each factor was also determined using ANOVA. In this study, the statistical analysis of the results and the optimization of the responses based on practical experimentation are described.

## 2 Materials and methods

### 2.1 Materials

Knitted fabrics having flexibility, sensorial and thermos-physiological comfort are the ideal choice for development of station suits for the next to skin wearing. The types of fibers chosen for this research were meta-aramid/Nomex fibers (*DuPont), OPAN carbon fibers (Zolteck),* modacrylic (Protex), *and FR Viscose fibers (China).* Since the application of the product is basically for fire fighters’ suits, the flame/fire retardant behavior was the primary requirement. All the selected fibers are inherently designed to have this property. Moreover, they are all commercially available in large quantities and at reasonable prices. Therefore, their blends and combination with different knitted structures were chosen for optimization of flame retardance as well as comfort performance in the station suits. The blend ratios were selected based on commercial blend compositions in these materials.

**Fig. 1:**
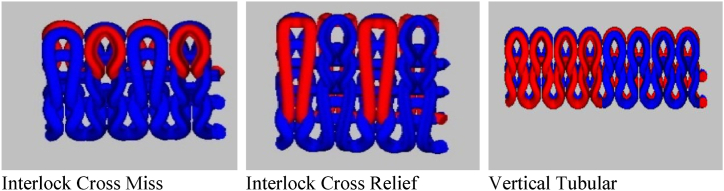
Structures of knitted fabrics.

Ring spun yarns of (30/1 Ne)/20 Tex were developed from different blend ratios that were selected from previous finding [13]. Three interlock knit fabric structures based on the previous findings were used for the development of fire-resistant fabrics as these structures exhibited better performance and comfort properties [14]. All knitted fabric samples were produced using Circular Interlock Knitting machine FUKUHARA LDR-L (Japan) available in knitting lab of National Textile University, Pakistan. This machine has a diameter of 75 cm and the gauge 20(E). The stitch length used for the development of all samples were set at 0.30 ± 0.02 cm, tightness factor of 14.7 ± 0.1 and tension was kept as constant for all samples as well. The structures developed on Wise Tex software are shown in Fig. 1.

### 2.2 Methods

#### 2.2.1 Experimental design

To investigate the performance characteristics of the FR fabrics, two control factors, e.g., the fiber blend % and knitted structure, were selected on the basis of literature review. The design of the experiments (DOE) is shown in Table 1.

**Table 1:** Factors and their levels/DOE.

**Factors**	**Levels**				
	1	2	3	4	5
Fiber Blend % (A)	100 % Nomex	Nomex 50 %: Carbon 50 %	Nomex 30 %: Protex 70 %	Nomex 70 %: Carbon 30 %	Nomex 70 %: Viscose 30 %
Knit fabric Structure (B)	Cross Miss	Cross Relief	Vertical Tubular	–	–

A total of fifteen knitted fabric samples were produced using four inherently FR fibers as mentioned in Table 2. A customized Taguchi design was used with 15 runs to conduct the experiment. Three control samples of 100 % Nomex were also produced in all knitted structures. All the laboratory tests were performed with respect to FR performance and comfort properties against two factors.

**Table 2:** Details of experiments.

**Sample code**	**Fiber%**	**Structure**
S1	100 % N	Cross Miss
S2	100 % N	Cross Relief
S3	100 % N	Vertical Tubular
S4	50 N/50C	Cross Miss
S5	50 N/50C	Cross Relief
S6	50 N/50C	Vertical Tubular
S7	70P/30 N	Cros Miss
S8	70P/30 N	Cross Relief
S9	70P/30 N	Vertical Tubular
S10	70 N/30C	Cros Miss
S11	70 N/30C	Cross Relief
S12	70 N/30C	Vertical Tubular
S13	70 N/30 V	Cros Miss
S14	70 N/30 V	Cross Relief
S15	70 N/30 V	Vertical Tubular

∗N=Nomex, C=Carbon, V=Viscose Rayon, P=Protex.

The specific reason for conducting the experiment using customized Taguchi design with two factors in this study, despite the usual application with three factors, is due to the nature of the study's experimental design. The study focused on optimizing the performance characteristics of fire-resistant fabrics by analyzing two main control factors: fiber blend percentage and knitted structure. These factors were chosen based on their significant impact on fabric properties such as flammability, comfort, and mechanical performance.

By selecting these two critical factors, the customized Taguchi design was employed to efficiently explore the combinations of fiber blends and knitted structures while minimizing the number of experimental runs. This allowed for a comprehensive evaluation of the effects of these two factors on fabric properties without the need for additional factors that may not be as relevant to the study's objectives.

### 2.3 Laboratory testing

#### 2.3.1 Physical properties

The Physical properties, such as areal density and thickness, were determined according to the standard test methods ASTM D 3776 and ASTM D1777, respectively [15,16].

The mechanical properties of the knitted fabric samples, i.e., the bursting strength and pilling resistance, were determined according to the standard test methods ISO 12938-2 and ISO 12945-2, respectively [17,18].

The bursting test measures the strength by applying hydraulic pressure through a rubber diaphragm on a circular area of the sample under test. A digital indicator was used to measure the force needed to burst the sample. It ranges from 0 to 500 KPa. The sample size was 15 × 15 cm. The tests were repeated for 10 samples of each type and the mean was calculated.

Pilling resistance was tested by using Martindale abrasion tester. It is a testing instrument that is used to assess the abrasion resistance and fabric pilling. Abrasion resistance is how resistant a fabric is against other materials after experiencing constant friction. 10,000 abrasion cycles were used for each sample. The sample size was 12 × 12 cm. The tests were repeated for 10 samples of each type and the mean was calculated. After the test, the pilled sample was compared to standard templates for assigning a grade as per ISO 12945-2 [18].

#### 2.3.2 Thermo-physiological comfort properties

The thermo-physiological comfort properties of the fabric samples were tested as per standard test methods. Air permeability plays a vital role in deciding comfort of fabric materials. The air permeability test was performed on the instrument Air permeability tester M021A (SDL ATLAS, South Carolina, USA) in accordance with the standard method ASTM-D-737 [19]. Thermal resistance of the fabrics is a very important aspect of the thermo-physiological comfort of the station suit materials. Thermal resistance was measured using instrument named Alambeta Thermolab (Sensora, Liberec, Czech Republic) according to standard test method ISO-11092 [20]. Overall moisture management capacity (OMMC) analyses the management of liquid moisture transport. To investigate the moisture management properties of fabric materials, moisture management tester MMT (SDL ATLAS, South Carolina, USA) was utilized. The test was done according to the standard test method AATCC-195.OMMC [21]. The test comprises the measurement of wetting time, maximum wetted radius, one way transport index, absorption rate and spreading speed of the liquid moisture. The tests were repeated 10 times for each sample and the average was reported.

#### 2.3.3 Tactile comfort

Tactile comfort is the subjective evaluation of the human sensation to the fabric materials. Touch/tactile comfort properties were tested using the instrument Phabrometer-3 fabric evaluation system (Nu Cybertek, Inc. Davis, USA) following the standard test method AATCC TM 202:2014 [22]. It is one of the most recently developed instruments for the evaluation of sensorial comfort. 100 % Nomex was used as the control sample and all other samples were compared with it.

#### 2.3.4 Dimensional stability

Dimensional stability of fabric samples was tested according to standard testing procedures directed in the standard method ASTM D 2594-99a [23]. The stretch and recovery ability are very important for knitted structure. Stretching is also directly linked to the comfort of a garment, as it relates to the ease of wearing a garment. The optimum level of stretchiness is a basic requirement in knitted fabrics. Poor stretching results in a poor fit, leading to performance shortcomings such as discomfort. Stretch measurements of all compression stockings were performed on a CETME Attrezzature Per Calzific instrument from Reggio Em., Italy, as per ASTM D 2594. The stretch is the amount of extension of the fabric under a determined force, while the elasticity is the fabric's recovery ability after stretching, which determines the dimensional stability of an elastic garment.

The stretch percentages were calculated using Equation (1):

(1) Fabricstretch%=(B–A)/A×100

where A is the original distance between marked points prior to the application of tension, B is the distance between benchmark points on the specimen under tension.

For the calculation of the recovery percentages, Equation (2) was used:

(2) $$Fabric\;recovery\;\%=\frac{B\;–\;D}{B-A}\times 100$$

where A is the original distance between marked points prior to the application of tension, B is the distance between benchmarks on the specimen under tension and D is the distance between benchmarks after the release of tension.

The tests were repeated 10 times for each sample and the average was reported.

Growth% was calculated as: Stretch% - Recovery% (3)

#### 2.3.5 Flammability test

Vertical flammability test was performed to analyze the burning behavior, melting, and dripping behavior of specimens. The test was performed on vertical flammability tester model number M233B. The vertical flammability tests were performed using standard test method ASTM D 6413 [24]. This is a standard test method to analyze the fire resistance of textiles. In this test burning behavior, char length, after flame time and afterglow time are measured. The char length is the length burnt after flaming. The after-flame time indicates the time taken to put off the flame, after the flame is extinguished. The after-glow time again shows the flameless glow after the fire has been put off. All the specimens were prepared in standard size mentioned in the test method (300 m × 80 mm) and flammability was measured under controlled laboratory conditions. Flame height was kept at 3.9 cm and for 12 s.

#### 2.3.6 Steady state (convective & radiant) heat resistance

Steady state (convective & radiant) heat resistance test was conducted to predict the thermal protective performance of fabrics. This test was conducted to predict the thermal protective performance of fabric materials according to the standard testing method ASTM F2700-08 [25]. The instrument was equipped with 12 quartz rods for heating purpose. The thermocouple was used to determine the temperature gradient. It measures convective and radiant heat time intervals as well as convective and radiant heat time gradients.

### 2.4 Taguchi optimization approach of multi-response by GRA

Optimization techniques were employed to find the optimal parameters that yield the best response variables. The Taguchi method was also used for the optimization of process parameters. In general, Taguchi is employed to optimize a single factor by the S/N ratio. This is the ratio of the signal/desirable value to the noise/undesirable value. Based on the targeted responses, signal-to-noise ratios can be chosen for any of three modes: larger is better, smaller is better, and nominal is better [[26], [27], [28], [29], [30]].

(4) $$SN\;ratio\;\left(Lower-the-better\right)=-10\;\log\frac{1}{n}\sum_{i=1}^{n}\frac{1}{y^{2}}$$

(5) $$\text{SN}\;\text{ratio}\;\left(\text{higher}-\text{the}-\text{better}\right)=-10\;\log\frac{1}{n}\sum_{i=1}^{n}y^{2}$$

(6) $$\text{SN}\;\text{ratio}\;\left(\text{nominal}-\text{the}-\text{better}\right)={-10\;\log\left(\bar{y}-y_{T}\right)}^{2}+Var\left(y\right)$$

Unit of S/N ratio is the same as of the response. For the comparison purpose unit-free normalized S/N ratios were obtained, which were applied in multi-response optimization analysis. The normalized equation for S/N ratios lower-the-better, higher-the-better, and nominal-the-better respectively are as follows;

(7) $$Normalized\;S/N\;ratio\;\left(Lower-the-better\right)=\frac{{SN}_{ij}^{\max}-{SN}_{ij}}{{SN}_{ij}^{\max}-{SN}_{ij}^{\min}}=Z_{ij}$$

(8) $$Normalized\;S/N\;ratio\;\left(\text{higher}-\text{the}-\text{better}\right)=\frac{{SN}_{ij}-{SN}_{ij}^{\min}}{{SN}_{ij}^{\max}-{SN}_{ij}^{\min}}=Z_{ij}$$

(9) $$Normalized\;S/N\;ratio\;\left(\text{nominal}-\text{the}-\text{better}\right)=\frac{\left|{SN}_{ij}-To\right|-{\left|{SN}_{ij}-T\right|}^{\min}}{{\left|{SN}_{ij}-T_{o}\right|}^{\max}-{\left|{SN}_{ij}-T_{o}\right|}^{\min}}=Z_{ij}$$

For the further processes normalized S/N ratios were applied to represent the multi-responses into single response by employing Taguchi-based multi-response optimization technique. In the present study, GRA was applied to all the response data to optimize the responses and rank the samples accordingly. Hence, to carry out multi-response optimization of FR fabric performance, grey relational normalization, the grey relational coefficient and the grey relational grade were calculated. The stepwise procedure of the GRA was performed as described by other researchers [[26], [27], [28], [29], [30]].

The objective of this study was to either obtain higher values or lower values for some responses. Therefore, the quality characteristics required for the present study were classified as follows: larger is better; or smaller is better.

### 2.5 Analysis of variance (ANOVA)

One-way ANOVA was performed with reference to both the factors i.e., fiber blend % and the fabric structure. ANOVA is normally used to assess the effects of factors on the responses. A larger F value indicates the significant effect of a factor on performance/response. The correlation coefficient R-square (R^2^) represents the validity of the fitted model. Higher R^2^ values signify the goodness of fit of the developed model [31,32].

ANOVA was performed on the data to determine the significance of the results. The significance level (α) selected for the study was 0.05, and the analysis was conducted using the Minitab Statistical software package. ANOVA was employed to the values of grey grade values of all the responses, to identify the significant factors for which the p-value was less than 5 %.

## 3 Results and discussion

### 3.1 Effects of different factors on all the responses obtained experimentally

**Fig. 2:**
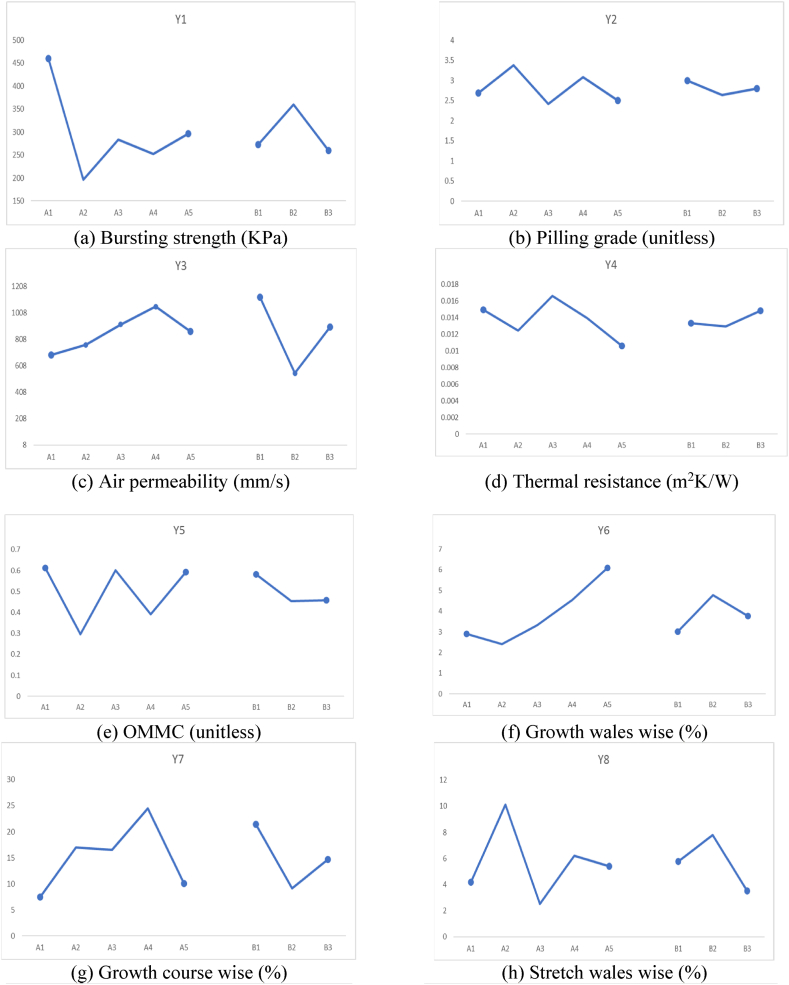

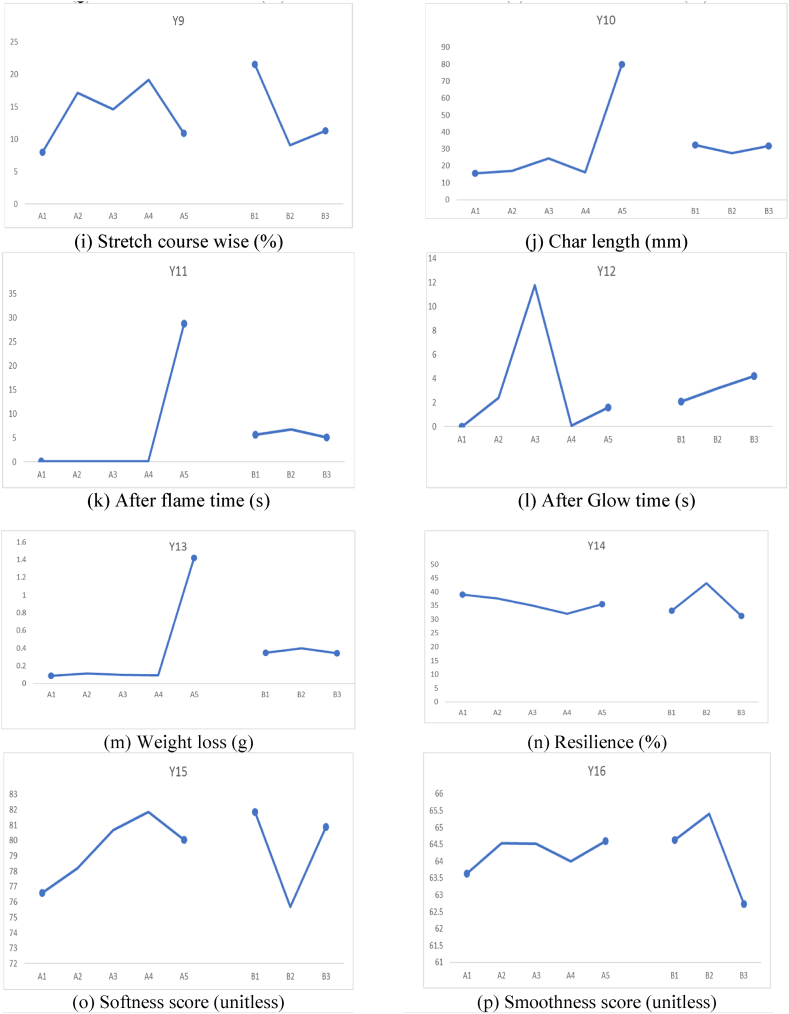

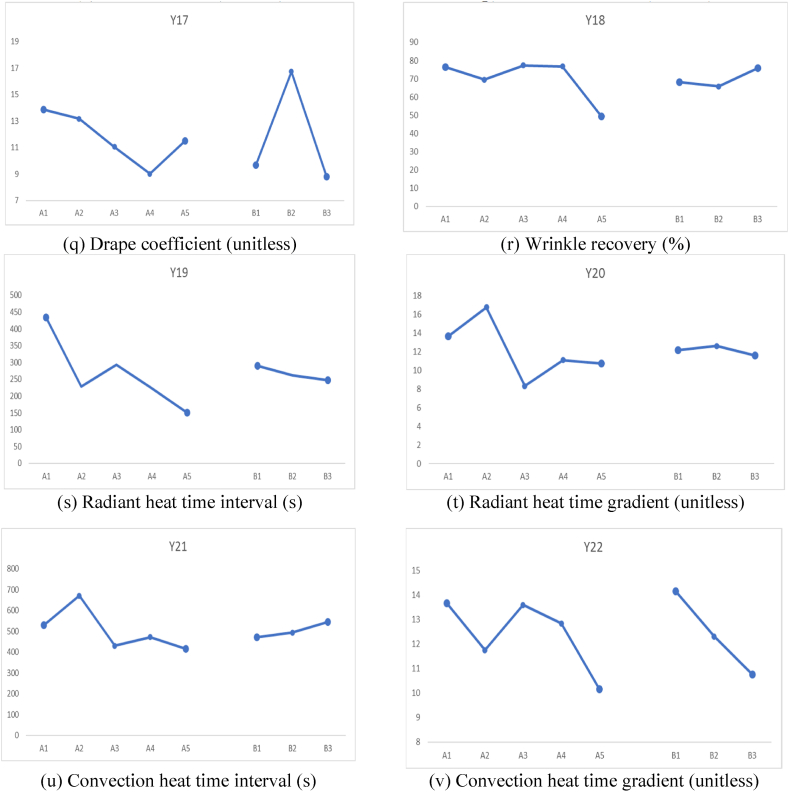
Effects of different factors on all the responses.

The effects of different factors on all the responses including mechanical properties, comfort properties, dimensional stability, and flammability are shown in Fig. 2.

It can be observed from Fig. 2(a) that the bursting strength was maximum for 100 % Nomex sample. It can be attributed to the excellent mechanical properties of the Nomex fibers which is a well-known fact. On the other hand, minimum bursting strength was observed for the sample with Nomex 50 %: Carbon 50 % composition. It can be due to the brittle nature of the carbon fibers which leads to lower bursting strength. In all other fiber combinations, as the proportion of Nomex fiber increases, the bursting strength also increases.

With respect to the knitting structure, the Cross Relief structure shows maximum bursting strength. This is mainly due to the maximum interlocking of the loops and a robust construction.

From Fig. 2(b), the effect of fiber composition on pilling resistance can be observed. It was observed that the blends of Carbon fiber resulted in higher pilling resistance as compared to other fiber blends. This is because, pilling is a surface phenomenon, and the carbon fibers have excellent surface resistance to wear and tear. The pilling resistance was minimum for the Cross Relief structure as it offers longer floats of yarn on the surface which are subject to wear due to abrasion. On the other hand, Cross Miss and Vertical tubular structures, offer curved loops for the abrasion which result in lower wear on the surface.

Fig. 2(c) shows that, Nomex 70 %: Carbon 30 % blend composition resulted in highest air permeability. This means that the specific combination resulted in macro pores facilitating easier passage for air. 100 % Nomex showed the minimum air permeability indicating absence of macro pores. It seems that Nomex 70 %: Carbon 30 % combination results in a loose construction of the blended yarn. With the different knit structures, it was observed that the Cross Relief structure known for longer yarn floats resulted in the minimum air permeability. This is due to the cohesion of the longer yarn floats which block the bigger pores and thus do not allow easy passage for air.

It is visible from Fig. 2(d) that, Nomex 30 %: Protex 70 % resulted in highest thermal resistance. It is well known that Protex is a modified acrylic fiber with wool-like properties. It can entrap micro air pockets like wool fiber and offer excellent thermal resistance. Otherwise, carbon and viscose blends with Nomex resulted in reduced thermal resistance due to lower micro porosity.100 % Nomex also resulted in a reasonably good thermal resistance owing to porosity of the yarn structure. The Cross Miss and Vertical tubular structures are compact, and they can entrap micro pores of air. Thus, they resulted in higher thermal resistance than Cross Relief structure which rather holds bigger pores that cannot offer effective thermal resistance.

It was observed from Fig. 2(e) that, the OMMC of the samples were inferior when carbon fibers were used in the blend. It can be due to the poor water absorption capacity of highly crystalline carbon fibers. The other fiber combinations show higher OMMC. The Cross Miss structure resulted in the best OMMC owing to the micro pores and the capillary action offered by these pores. The vertical tubular structure also resulted in reasonably good OMMC. However, the Cross Relief structure resulted in minimum OMMC due to absence of enough capillary action.

The wale wise Growth% is shown in Fig. 2(f). It shows that the Nomex 50 %: Carbon 50 % blend resulted in minimum Growth%. It means that the specific composition resulted in excellent recovery after removal of the tension. This is a very good sign for dimensional stability of a garment. On the other hand, the Nomex 70 %: Viscose 30 % blend showed maximum Growth indicating the poorest dimensional stability. This is due to the viscose component which shows poorer elastic properties. Among the different knitted structures, the Cross Relief structure showed highest wale wise Growth% due to lower recovery from stretch. The other two structures (Cross miss and Vertical Tubular) involved denser loops and thus are more elastic.

The course wise Growth% depicted in Fig. 2(g) indicate the complete opposite trend. It is due to the fact that a length wise extension is accompanied with a width wise shrinkage. Thus, the Carbon blended samples show higher course wise Growth%. Similarly, the Cross Relief structure resulted in minimum Growth in the course direction.

Fig. 2(h) indicates that the Carbon fiber blended samples show higher Stretch% as compared to other blends. This can be attributed to the smoother and slippery surface of carbon fibers, which does not show enough restraint under tension. On the other hand, fibers like Nomex, Protex and Viscose are highly crimped and resist any deformation more effectively. Among the structures involved, the Cross Relief structure employes longer floats and thus extends easily. Whereas the other two structures are denser and do not stretch as much.

The course wise stretch also is affected by the surface nature of the carbon fibers and shows similar trend in Fig. 2(i). However, the amount of Stretch% in the course direction is higher than Stretch% in the wale direction due to higher loop density. Further, the Stretch% of Cross Miss structure is the highest owing to the dense structure and the intermingled loop geometry.

Fig. 2(j–m) show the flammability related responses of the samples with respect to fiber blend composition and knitted structure. The char lengths shown in Fig. 2(j) indicate that, all the blends except Nomex 70 %: Viscose 30 % shown much lower char length indicating excellent flame resistance. The Viscose is a cellulosic origin fiber and thus cannot resist the flame as much as Nomex, carbon or Protex. Among the knitted structures, there is no significant difference in the char length. It is because, the flammability of more of a fiber property rather than the property of the macro structure.

Fig. 2(k) shows the after-flame time of all the different blends and structures. It indicates a zero after flame time for all the samples except Nomex 70 %: Viscose 30 % blend. This supports the earlier observation of poorer flame resistance offered by the Viscose fibers. The after-flame time is also not significantly different among the different knitted structures.

The after-glow time depicted in Fig. 2(l) shows a quite different trend. The Nomex 30 %: Protex 70 % blend shows a significantly higher after-glow time as compared to other blends. It can be attributed to the Protex (modacrylic fibers) which show a flameless glow after the fire has been put off. The Vertical Tubular structure shows the highest after-glow time among all the knit structures. This can be due to the tubular channel, which facilitates flameless glowing due to availability of air.

The weight loss depends on the flammability and combustion of the samples. The Viscose blend resulted in significantly higher weight loss as compared to other blend compositions as shown in Fig. 2(m). The other samples showed negligible weight loss. The knit structure did not affect the weight loss significantly, as it is an intrinsic behavior of the fibers rather than the macro structural behavior.

Fig. 2(n–r) depict the comfort related aspects of the different blends and the structures. There is no significant affect of the blend proportions on the resilience shown in Fig. 2(n). This is due to the fact that fabric resilience is a macroscale property mainly due to the structure. It can be observed that the Cross Relief structure exhibited higher resilience as compared to the Cross Miss and Vertical Tubular structure. It is closely related to the Stretch of the fabrics.

The softness shown in Fig. 2(o), indicates that Nomex 70 %: Carbon 30 % and Nomex 70 %: Viscose 30 % resulted in a slightly higher softness as compared to the other compositions. However, the values are not significantly different. The Cross Miss and Vertical Tubular structures are softer than the Cross Relief structure. It can be due to the loop structures in these fabrics. The smoothness is not very significantly different for the different fiber blends as shown in Fig. 2(p). Since it is mainly a surface property, it mainly depends on the knitted geometry. The Vertical Tubular structure resulted in a relatively rough surface and therefore, a lower smoothness than the other structures. The drape coefficient is shown in Fig. 2(q). As it mainly depicts the hanging behavior resulting from stiffness and weight, it was observed to be the lowest for Nomex 70 %: Carbon 30 % which also proved to be mechanically weaker as compared to other samples. The Cross Relief structure involving longer yarn floats resulted in higher stiffness and higher drape coefficient as compared to the other structures.

The wrinkle recovery is the ability to overcome irregular folds. It depends on the stiffness of the material. The Nomex 70 %: Viscose 30 % showed a much lower wrinkle recovery as compared to other blends as shown in Fig. 2(r). This is attributed to lower stiffness of the Viscose fibers. No significant difference was observed among the different structures.

Fig. 2(s–v) depict the steady sate heat resistance. The radiant heat time interval (Fig. 2(s)) was observed to be highest for 100 % Nomex, which is the most resistant to flammability. There was no significant difference among the samples. Fig. 2(t) shows the radiant heat time gradient which is the highest for Nomex 50 %: Carbon 50 % blend and no significant effect of the structure was observed. The convection heat time interval (Fig. 2(u)) shows a different trend than the radiant heat time interval since the mechanism of heat transfer is different. Nomex 50 %: Carbon 50 % showed highest convection heat time interval indicating higher resistance to convective heat transfer. The structure remained ineffective in this case as well. Further the convection heat time gradient (in Fig. 2(v)) shows that 100 % Nomex and Nomex 30 %: Protex 70 % are the best against convective heat loss. The Cross Miss structure is the best with respect to convective heat resistance. The Vertical Tubukar structure cannot prevent convection that effectively.

The dependencies of various experimental responses on the input variables can be summarized as shown in Table 3.

**Table 3:** The dependencies of various experimental responses on the input variables.

***Input parameters***	***Fiber blend %*** *(Dependent significantly- ✔)* *(Not dependent significantly-* ✗*)*	***Knitting structure*** *(Dependent significantly- ✔)* *(Not dependent significantly-* ✗*)*
Properties		
Bursting Strength (BS)	*✔*	*✔*
Pilling Resistance (PR)	*✔*	*✔*
Air Permeability (AP)	*✔*	*✔*
Thermal Resistance (TR)	*✔*	*✔*
Overall Moisture Management Capacity (OMMC)	*✔*	*✔*
Fabric Growth Wales wise (GW)	*✔*	*✔*
Fabric Growth Course Wise (GC)	*✔*	*✔*
Fabric Stretch Wales Wise (SW)	*✔*	*✔*
Fabric Stretch Course Wise (SC)	*✔*	*✔*
Char Length (CL)	*✔*	✗
After Flame Time (AFT)	*✔*	✗
After Glow Time (AGT)	*✔*	*✔*
Weight Loss (WL)	*✔*	✗
Resilience (%)	✗	*✔*
Softness Score (SfS)	✗	*✔*
Smoothness Score (SS)	✗	*✔*
Drape Coefficient	*✔*	*✔*
Wrinkle Recovery (WR)	*✔*	✗
Radiant Heat Time interval (CHTI)	*✔*	✗
Radiant Heat Temperature Gradient (RHTG)	*✔*	✗
Convective Heat Time interval (CHTI)	*✔*	✗
Convective Heat Temperature Gradient (CHTG)	*✔*	*✔*

### 3.2 Analysis of variance (ANOVA) of the responses

The ANOVA results for all the physical, mechanical and comfort related responses are given in Table 4.

**Table 4:** ANOVA results for all the physical, mechanical and comfort related responses.

**Properties**	**Fiber Blend %**			**Fabric Structure**		
	*p value*	F Value	R-sq	*p value*	F Value	R-sq
Areal Density	0.101	2.59	50.87	0.021	5.41	47.42
Thickness	0.314	1.36	35.29	0.004	9.31	60.81
Bursting Strength (BS)	0.003	8.48	77.22	0.26	1.51	20.12
Pilling Resistance (PR)	0.102	2.58	50.76	0.564	0.6	9.09
Air Permeability (AP)	0.003	8.48	20.79	0	17.15	74.08
Thermal Resistance (TR)	0.003	8.48	77.24	0.513	0.71	10.52
Overall Moisture Management Capacity (OMMC)	0.35	1.25	33.41	0.43	0.91	13.11
Relative Hand Value (RHV)	0.989	0.07	2.8	0	18.86	75.86
Softness Score (SfS)	0.421	1.07	29.94	0.002	11.39	65.49
Smoothness Score (SS)	0.903	0.25	9.11	0	23.15	79.41
Drape Coefficient	0.425	0.20	4.42	0	14.52	24.53
Wrinkle Recovery (WR)	0.85	0.33	11.62	0.708	0.36	5.59
Fabric Growth Wales wise (GW)	0.253	1.58	38.78	0.397	1	14.29
Fabric Growth Course Wise (GC)	0.145	2.18	46.57	0.086	3.04	33.61
Fabric Stretch Wales Wise (SW)	0.22	1.73	40.88	0.27	1.46	19.58
Fabric Stretch Course Wise (SC)	0.554	0.8	24.15	4.49	0.035	42.8

#### 3.2.1 Physical properties

From Table 4, the effect of the fiber blend percentage on the areal density of the samples was not significant, as *the p value* was greater than 0.05. The effect of fabric structure on areal density was significant, as *the p value* was 0.02, which was lower than 0.05. In this context fabric structure plays a vital role in deciding the real density of the fabrics because as the fabric structure changes, the percentage of different stitches e.g., knit, tuck and miss varies which result in variation in the compactness of the fabric. This ultimately varies the number of fibers per unit area of the fabric, and as a result there is prominent change in the areal density of the fabric by changing the fabric structure [33]. A similar trend was observed for the thickness of the samples. Therefore, it can be concluded that the areal density and thickness exhibit similar trends, as both are prominently affected by the fabric structure. Fabric areal density and thickness are directly related to each other. The correlation between fabric structure, its areal density and thickness is evident from the ANOVA.

#### 3.2.2 Mechanical properties

Table 4 clearly shows that the fiber blend % has a significant effect on the bursting strength of the samples. The *p value* of the bursting strength considering fiber blend% is 0.003. The significant effect of the fiber blend% on the bursting strength is because, it depends on the fiber properties e.g., its strength, elongation and tenacity. These results which illustrate that different fibers result in varying the mechanical properties of fabrics is in accordance with the previous findings [34,35]. 100 % Nomex fiber-based sample has highest bursting strength. This can also be seen in Fig. 2(a). The plots shown in Fig. 2(a–v) demonstrate the effects of both factors, i.e., the fiber blend % and the fabric structure, on the different responses.

For the case of the pilling resistance, the results are not significant, and no trends were observed, as shown in Fig. 2(b). This is because in case of pilling property the combined effect of fiber properties and fabrics characteristics is demonstrated. Moreover in case of blended yarns the combined effect of both fibers for example the frictional coefficient of fibers, scales on fiber surface, crimps etc. are also responsible for pilling behavior [36].Variation in the percentage and location of different stitch types result in changing the pilling behavior of resultant fabrics [37,38].

#### 3.2.3 Comfort properties

##### 3.2.3.1 Thermo-physiological comfort properties

Table 4 shows that the effect of the fiber blend % and fabric structure significantly affects the air permeability of the knitted fabric samples. Air permeability is influenced by the yarn characteristics, fibers composition as well as fabric structural characteristics like porosity of fabric structure, thickness, areal density etc. [39,40]. In the case of thermal resistance, it is evident that the fiber blend % has a dominant effect on the thermal resistance of the knitted fabric samples (*p* = 0.003). This is because the fiber intrinsic properties affect the thermal resistance which involve fiber morphological characteristics like the scales present on the surface of wool fibers assist in insulation behavior [41]. Moisture management properties are not significantly affected by both the factors as p-value is higher than 0.05 for both the factors. All the comfort properties plots are shown in Fig. 2(c–e).

##### 3.2.3.2 Tactile comfort properties

The results of ANOVA (from Table 4) against tactile properties suggest that fabric structure has a dominant effect. The ANOVA results for the Relative Hand value (RHV) in Table 4 show that the fabric structure has a dominant effect on the Relative hand values of the samples. Similarly, p-value of smoothness and softness scores are lower than 0.05, which confirms the significance of the effect of fabric structure on tactile properties. Surface properties of knitted fabrics are influenced greatly by the structural attributes of the fabrics. It has been already found in the literature and also in this statistical analysis. These findings are in line with the work reported in literature*,* in which the Hand value of knitted fabrics were greatly influenced by the type of knit structure used in the development of fabrics [42,43].

#### 3.2.4 Dimensional properties

Overall, all the properties are not significantly affected by either factor. In all the results, the *p* value is much greater than 0.05, which means that the results are not statistically significant. Additionally, the lower F value also reveals that there is greater variation within the samples. It can be seen in Fig. 2(f–i). The lower R-square values also support the argument that the model is not fit. In this work, different blended yarns and knit structures were used so it seems difficult to analyze the effect of individual parameters. The combined effect of fibers in the blend also play a role along with fabric structural parameters.

#### 3.2.5 Flammability properties of fabric samples

**Fig. 3:**
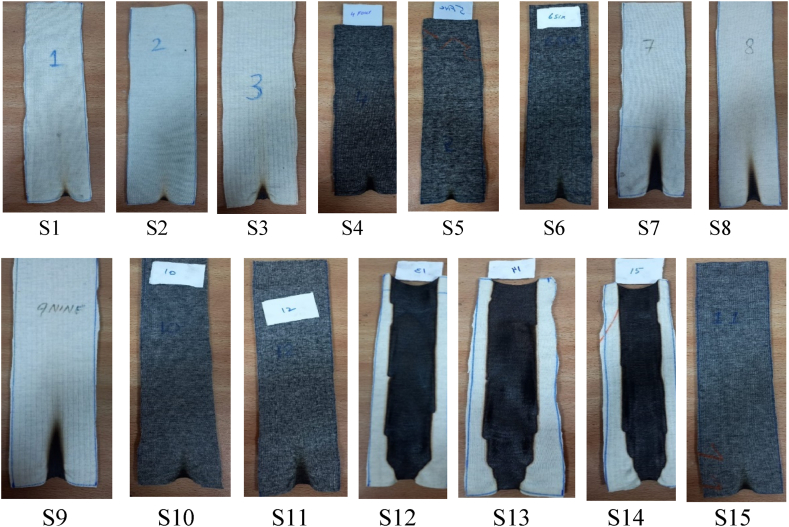
Samples after the vertical flammability test.

The samples were tested for vertical flammability, as shown in Fig. 3.

Table 5 shows that the fiber blend % has a significant effect on most of the flammability related responses. Char formation is the characteristic of the constituent fibers used in the production of samples. The chemical composition of the fibers play decisive role in the exhibition of their flammable behavior [44]. The ANOVA of Char length vs fiber blend percentage suggested that fibers had a significant effect on the char length of the samples, which was supported by the p-value, as shown in Table 5. Fiber blend % and fabric structure suggested that the *p-value* of the fiber blend percentage had a dominant effect on all the properties including weight loss, after-flaming time, after glowing time, while the fabric structure did not have a significant effect and can be seen in Fig. 2(g–j). The Burning behavior of fabrics is very much dependent on the type of fibers used in the development of fabrics. The pyrolyzing behavior and limiting Oxygen index of fibers are critical aspects involved in deciding the resultant burning mechanism of fabric samples. Sometimes the better blending of fibers in yarns exhibit synergistic effect that's why blend percentage has dominant effect in this case as well [45].

**Table 5:** ANOVA results of flammability and steady-state heat resistance responses.

**Flammability properties**	**Fiber Blend %**			**Fabric Structure**		
	***p value***	**F Value**	**R-sq**	***p value***	**F Value**	**R-sq**
Char Length (CL)	0	178.27	98.62	0.955	0.05	0.77
Weight Loss (WL)	0	177.57	98.61	0.985	0.01	0.24
After Flame Time (AFT)	0	120.14	97.6	0.976	0.02	0.41
After Glow Time (AGT)	0	20.19	88.98	0.811	0.21	3.43
Radiant Heat Time interval (CHTI)	0.011	4.02	83.57	0.441	0.41	4.71
Radiant Heat Temperature Gradient (RHTG)	0.166	2.03	44.77	0.95	0.05	0.85
Convective Heat Time interval (CHTI)	0.027	4.36	63.57	0.626	0.49	7.52
Convective Heat Temperature Gradient (CHTG)	0.459	0.98	28.22	0.153	2.2	26.86

The ANOVA results for steady-state heat resistance shown in Table 5 suggest that the fiber blend % has a major impact on the heat resistance of the fabric samples, which is confirmed by the lower *p*-value of the convective heat time interval. The *p-*value in the mentioned case was less than 0.05.

The flammability properties of a fabric are heavily influenced by the inherent fire-resistant properties of the fibers used in the blend rather than the macroscale constructional variations. In our study, the fiber blend (e.g., Protex, Nomex, Carbon, Viscose) played a dominant role in determining the fabric's flammability characteristics. These fibers have specific chemical compositions that directly impact properties like char formation, after-flame time, and after-glow time. Fabric structure, on the other hand, primarily affects mechanical and comfort-related properties but has a less direct influence on fire resistance as compared to the fiber blend composition.

### 3.3 GRA methodology

To achieve the optimal level of all the factors for the response variables, different techniques are available. The grey relational analysis (GRA) was used to effectively address the complicated interrelationships between multiple responses. Taguchi method can be utilized along with GRA for the optimization of responses. The Taguchi method was used in the conversion of multiple performance characteristics into single performance characteristics, which are called grey grades. The integration of the Taguchi method and GRA was proposed by researchers when a system has insufficient or unknown information, too much data or vagueness. Unknown information is often called grey. GRA has been found to be a quantitative approach. It is used for solving problems and optimizing responses. GRA also helps in sorting or ranking in the process of decision making as well as prediction [46].

In the present study, two factors are considered. The significance and influence of factors is decided by ANOVA and further optimization of the parameters was done using Grey Relational analysis as GRA is very beneficial in case of multi objective optimization. The customized Taguchi design was employed, and the average results of each run were taken into consideration in the analysis, as shown in Table 6 (Annexure).

Table 6. Customized Taguchi design with responses (Annexure).

The methodology of GRA discussed was employed step-by-step to get the results.

➢ The signal-to-noise (S/N) ratios of all the responses were computed by using equations (4), (5), (6) and given in Table 7 (Annexure). It measures the variation in the responses regarding the target value/quality characteristics either higher, lower or nominal the better. The S/N ratios were expressed as higher the better in case of Bursting strength, pilling resistance, comfort properties (thermo-physiological and tactile) and convection heat time and temperature. And lower is better in case of fabric growth and stretch, char length, after flame time, weight loss % and radiant heat time and temperature.

Table 7. S/N ratio value at all runs (Annexure).

➢ In this step, normalized S/N ratio values of each response were calculated using Eq.s (7), (8), (9)) according to the relevant characteristic to determine the GRA for the series of comparable characteristics. The results of normalized S/N rations of each response are given in Table 8 (Annexure).

Table 8. Normalized S/N ratio values at all runs (Annexure).

Quality loss functions by using the following eq. were evaluated.

(10) Δ = (quality loss) = |Z_o-Z_ij |

The S/N ratios are inversely proportional to the quality loss function given in Table 9 (Annexure). The maximization of the S/N ratio means minimizing the losses. Therefore, samples with higher S/N ratios and different responses are important to consider. The evaluation of the responses was performed by considering the grey grades. The response optimization was performed by the conversion of the optimized responses into grey grades. The absolute difference/quality loss function at all levels is given in Table 9 (Annexure).

Table 9. Absolute difference/Quality Loss function (Annexure).

Computation of the grey relational coefficient (GC) for the normalized SN ratio values was done. The grey relational coefficients for all runs are given in Table 10 (Annexure).

(11) $${\text{GC}}_{\text{ij}}=\frac{\Delta_{\min}+\delta \;\Delta_{\max}}{\Delta_{\text{ij}}+\delta \;\Delta_{\max}}$$

Where,

GC_ij_ = grey relational coefficient for the ith replicate of jth response, i = 1, 2, …,n and j = 1, 2, …, k.

Z_oj_ = optimized normalized value.

Z_ij_ = the ith normalized value of the jth response.

$\Delta_{\min}=$ minimum value of loss function Δ

$\Delta_{\max}=$ maximum value of loss function Δ

δ = coefficient of proportionality ranges 0≤δ≤1

Table 10. Grey relational Coefficients (Annexure).

➢ Based on the grey relational coefficients, grey grades were computed by taking average of the grey coefficients corresponding to each responses using Eq. (12). The ranking of the series of the grey grades for the optimized samples refer to the grey relational order, shown in Table 10.

(12) $$G_{j}=\text{average}\;\text{of}\;{\text{GC}}_{\text{ij}}=\frac{1}{m}\sum {\text{GC}}_{\text{ij}}$$

➢ Finally, the validity of GRA was examined by taking assigned ranks. Rankings of the samples were based on multiple responses depending on the grey grade. Higher grey values indicate a strong relationship between the parameters and responses. A higher grey grade indicates better performance and rank. The highest grey grade implies the most optimal process parameters which tells us how ideal performance by process variables. So, the samples were ranked best on the basis of grey grades. The grey grades and rankings are shown in Table 11.

**Table 11:** Grey grades values at all runs and rankings.

**Sample code**	**Grey grades**	**Rankings**
S1	0.714069155	10
S2	0.746058099	2
S3	0.716983872	8
S4	0.706025488	11
S5	0.736532938	3
S6	0.714982347	9
S7	0.720937026	6
S8	0.750233634	1
S9	0.694013058	13
S10	0.722207006	5
S11	0.697625787	12
S12	0.687457434	14
S13	0.730834534	4
S14	0.717614234	7
S15	0.664891366	15

The study employed Grey Relational Analysis (GRA) to optimize multiple responses by converting them into grey grades and ranking the fabric samples accordingly. The focus was on using the Taguchi method to calculate the S/N ratios for each run and then transforming these ratios into Grey Relational Grades to prioritize the responses. The mean of the S/N ratios or mean GRG values were not calculated explicitly because the optimization process focused on individual runs and their corresponding grey grades to rank the samples. The goal was to evaluate the performance of each sample rather than averaging the responses across all samples.

### 3.4 ANOVA for grey relational grade (GRG)

**Fig. 4:**
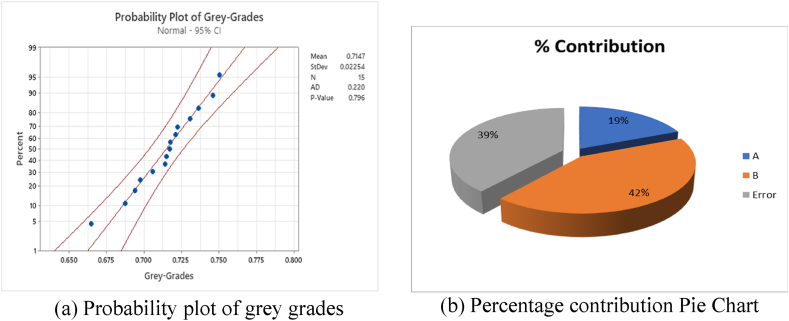
Contribution of factors in the GRG: (a) probability plot of grey grades and (b) percentage contribution of factors in the GRG.

To study the percentage contribution of each factor on FR fabric performance characteristics, an ANOVA was performed for GRG at the 95 % confidence level. In Table 12, the higher F test values for the fabric structure indicate that the fabric structure has a greater effect on the performance characteristics as compared to fiber blend %. The percentage contributions of both factors are shown in Table 12 and Fig. 4. A higher percentage contribution of the factor B, which is fabric structure, shows that the structure has a major contribution/impact on the numerous properties/responses of the FR fabric.

**Table 12:** ANOVA for grey relational grade (GRG).

**Factor**	**DF**	**Adj. SS**	**Adj.MS**	**F Test**	**Contribution (%)**
**A**	4	0.001339	0.000335	0.97	18.83
**B**	2	0.003008	0.001504	4.35	42.31
**Error**	8	0.002763	0.000345	–	38.86
**Total**	14	0.007110	–	–	–

In the Grey Relational Analysis (GRA) used for multi-response optimization, several different responses such as bursting strength, pilling grade, air permeability, thermal resistance, and others were aggregated into a single grey relational grade. Each of these responses is affected by different physical phenomena, and not all are influenced equally by fiber blend percentage. For instance, while fiber blend may significantly affect flammability and thermal resistance, it may not have as much impact on other properties like pilling resistance or smoothness. This introduces variability in the overall GRG, which is captured as error variance.

Normalized grey values are plotted in the probability plots shown in Fig. 4. The probability plots demonstrate that all the grey grades are normalized, and the normalized data lie within the normal region, which indicates the significance of the data. The normality of the data also indicated that further responses can be predicted based on the statistics and the ratios of the responses. The straight line in the middle is fitted distribution line and the line on the side are confidence intervals for the individual percentiles. Points lie close to the fitted distribution line, implying that the normal distribution is good fit to the data.

The statistical analysis, particularly the Analysis of Variance (ANOVA) results, indicated significant effects of the selected factors on the fabric properties. These findings provided confidence that the identified optimal combinations were likely effective. The focus on significant factors and their levels suggested that there is already a solid basis for the findings, reducing the immediate need for confirmation experiments in the initial study. Future work will involve performing confirmation experiments to test the robustness of the optimized conditions, providing a broader context for the findings and their applicability in practical settings.

## 4 Conclusions

The present study is an approach of integrating the Taguchi method and the multi-response GRA technique to evaluate the responses e.g., mechanical performance, dimensional stability, fire protection and comfort properties of the flame-resistant fabric materials in the practical field. The significance of each contributing factor was investigated by statistical analysis. ANOVA was applied to investigate the effect of two factors i.e. fiber blend % and knitted fabric structure on different properties/responses such as bursting strength, thermal resistance, convective heat resistance and flammability etc. These were significantly affected by the fiber blend, while other responses, such as areal density, thickness, RHV, hardness and smoothness, were affected by the fabric structure. It was noted that the air permeability and OMMC content were affected by the combination of the fiber blend and fabric structure.

It was concluded that sample (S8), composed of 70 % Protex/30 % Nomex blend with a Cross-Relief structure, was ranked 1st, as it exhibited the best optimized performance very close to the ideal condition based on the grey grades. This sample exhibited the best flammability properties in addition to relatively good comfort properties. Sample S2, composed of 100 % Nomex (control) with a Cross Relief structure, was ranked 2nd^,^ and sample S5, composed of 50 % Nomex/50 % Carbon with a Cross Relief structure, was adjudged 3rd. From this evaluation, it was concluded that the Cross Relief structure is the optimum fabric for all aspects of a firefighter's clothing. It was also concluded via ANOVA of GRA that the effect of the factor knitted structure on multiple performance characteristics was most significant, i.e., 42.3 %. This research presented an effective method for the optimization of the FR fabrics with multi response parameters based on GRA. The findings of this study can be used for the optimization of firefighters' clothing and the development of similar protective clothing materials.

## CRediT authorship contribution statement

**Hafsa Jamshaid:** Writing – original draft, Supervision, Project administration, Methodology, Investigation, Funding acquisition, Formal analysis, Data curation, Conceptualization. **Awais Ahmad Khan:** Writing – original draft, Methodology, Investigation, Formal analysis, Data curation, Conceptualization. **Rajesh Kumar Mishra:** Writing – original draft, Supervision, Resources, Project administration, Methodology, Investigation, Funding acquisition, Formal analysis, Data curation, Conceptualization. **Naseer Ahmad:** Writing – original draft, Project administration, Methodology, Investigation, Formal analysis, Conceptualization. **Vijay Chandan:** Writing – original draft, Validation, Resources, Methodology, Investigation, Formal analysis, Data curation, Conceptualization. **Viktor Kolář:** Writing – original draft, Methodology, Investigation, Formal analysis, Data curation, Conceptualization. **Miroslav Müller:** Writing – original draft, Supervision, Project administration, Funding acquisition, Formal analysis, Data curation, Conceptualization.

## Data availability statement

Data is provided within the manuscript or supplementary information files.

## Ethical approval

This work does not require ethical approval, as no experiment is being carried out on living beings.

## Declaration of competing interest

The authors declare that they have no known competing financial interests or personal relationships that could have appeared to influence the work reported in this paper.
