# Supplementary material for: Taguchi Grey relational analysis (GRA) based multi response optimization of flammability, comfort and mechanical properties in station suits
Source: Heliyon. 2025 Feb 7;11(4):e42508. doi: 10.1016/j.heliyon.2025.e42508 (PMC11869111; doi:10.1016/j.heliyon.2025.e42508)
Supplement: Multimedia component 1 [file mmc1.docx]

**Table 6.** Customized Taguchi design with responses.

| **Responses** | **Bursting strength**  **(KPa)** | **Pilling grade** | **AP (mm/s)** | **TR (m^2^K/W)** | **OMMC** | **GW%** | **GC%** | **SW%** | **SC%** | **CL (mm)** | **AFT (s)** | **AGT (s)** | **WD (g)** | **R (%)** | **SfS** | **SS** | **DC** | **WR** | **RHTI (s)** | **RHTG** | **CHTI (s)** | **CHTG** |
| --- | --- | --- | --- | --- | --- | --- | --- | --- | --- | --- | --- | --- | --- | --- | --- | --- | --- | --- | --- | --- | --- | --- |
| **Sample code** | **Y1** | **Y2** | **Y3** | **Y4** | **Y5** | **Y6** | **Y7** | **Y8** | **Y9** | **Y10** | **Y11** | **Y12** | **Y13** | **Y14** | **Y15** | **Y16** | **Y17** | **Y18** | **Y19** | **Y20** | **Y21** | **Y22** |
| S1 | 5 | 2.48 | 1112.25 | 0.02 | 0.60 | 2.38 | 12.50 | 2.50 | 11.28 | 28.50 | 0.10 | 0.01 | 0.07 | 36.16 | 79.75 | 64.89 | 12.06 | 70.00 | 585.00 | 6.75 | 519.25 | 15.75 |
| S2 | 562.75 | 2.45 | 274.00 | 0.01 | 0.62 | 3.74 | 6.36 | 6.40 | 6.27 | 8.55 | 0.10 | 0.01 | 0.09 | 48.54 | 71.40 | 64.58 | 19.63 | 79.34 | 282.50 | 18.50 | 441.25 | 13.75 |
| S3 | 410.00 | 3.13 | 686.38 | 0.02 | 0.62 | 2.63 | 3.54 | 3.68 | 6.32 | 9.38 | 0.10 | 0.01 | 0.10 | 32.07 | 78.68 | 61.43 | 9.96 | 79.83 | 435.00 | 15.75 | 626.50 | 11.50 |
| S4 | 196.25 | 3.13 | 937.25 | 0.01 | 0.28 | 0.01 | 27.41 | 15.05 | 31.27 | 16.88 | 0.10 | 1.43 | 0.11 | 35.47 | 80.44 | 65.01 | 11.52 | 67.27 | 242.50 | 22.75 | 522.75 | 9.50 |
| S5 | 237.00 | 3.13 | 547.38 | 0.01 | 0.16 | 3.56 | 4.94 | 12.46 | 10.05 | 14.38 | 0.10 | 2.24 | 0.11 | 45.53 | 73.54 | 65.70 | 18.78 | 71.24 | 252.50 | 15.75 | 712.50 | 13.00 |
| S6 | 154.75 | 3.88 | 813.38 | 0.01 | 0.45 | 3.68 | 18.73 | 2.86 | 10.10 | 20.00 | 0.10 | 3.50 | 0.13 | 31.60 | 80.57 | 62.92 | 9.20 | 70.00 | 190.00 | 11.75 | 777.50 | 12.75 |
| S7 | 261.75 | 3.00 | 1181.00 | 0.01 | 0.61 | 3.71 | 22.54 | 2.58 | 27.44 | 24.75 | 0.10 | 7.63 | 0.10 | 31.60 | 83.28 | 64.45 | 8.34 | 82.09 | 247.50 | 10.25 | 495.00 | 14.00 |
| S8 | 346.25 | 2.13 | 603.50 | 0.02 | 0.61 | 3.72 | 14.43 | 2.55 | 5.08 | 21.00 | 0.10 | 11.63 | 0.09 | 42.35 | 77.27 | 66.16 | 16.29 | 80.02 | 335.00 | 6.00 | 399.00 | 14.75 |
| S9 | 242.75 | 2.13 | 976.25 | 0.02 | 0.59 | 2.50 | 12.50 | 2.45 | 11.30 | 26.99 | 0.10 | 16.19 | 0.10 | 31.37 | 81.48 | 62.94 | 8.57 | 70.00 | 297.50 | 8.75 | 399.00 | 12.00 |
| S10 | 223.25 | 3.50 | 1313.00 | 0.01 | 0.64 | 6.25 | 34.50 | 2.50 | 29.92 | 12.13 | 0.10 | 0.10 | 0.08 | 29.61 | 83.47 | 64.21 | 7.30 | 73.44 | 197.50 | 12.75 | 399.00 | 16.00 |
| S11 | 311.75 | 2.88 | 751.75 | 0.01 | 0.23 | 3.63 | 16.27 | 9.91 | 12.46 | 14.00 | 0.10 | 0.10 | 0.10 | 36.73 | 79.55 | 64.58 | 12.33 | 75.78 | 282.50 | 10.75 | 515.00 | 11.25 |
| S12 | 221.00 | 2.88 | 1096.75 | 0.02 | 0.30 | 3.71 | 22.67 | 6.25 | 15.10 | 22.00 | 0.10 | 0.10 | 0.10 | 29.55 | 82.52 | 63.22 | 7.41 | 81.19 | 192.50 | 9.75 | 502.50 | 11.25 |
| S13 | 276.00 | 2.88 | 1091.00 | 0.01 | 0.79 | 2.75 | 10.28 | 6.25 | 7.75 | 80.00 | 27.85 | 1.38 | 1.38 | 32.51 | 82.41 | 64.62 | 9.13 | 47.58 | 180.00 | 8.25 | 427.50 | 15.50 |
| S14 | 343.50 | 2.63 | 580.75 | 0.01 | 0.65 | 9.19 | 3.81 | 7.57 | 11.28 | 80.00 | 33.38 | 2.00 | 1.61 | 42.66 | 76.69 | 66.05 | 16.61 | 22.78 | 152.50 | 12.00 | 402.50 | 8.75 |
| S15 | 268.25 | 2.00 | 927.50 | 0.01 | 0.34 | 6.31 | 16.00 | 2.39 | 13.72 | 80.00 | 25.10 | 1.44 | 1.28 | 31.47 | 81.06 | 63.12 | 8.80 | 77.70 | 120.00 | 12.00 | 420.00 | 6.25 |

**Table 7.** S/N ratio value at all runs.

| **Responses** | **Y1** | **Y2** | **Y3** | **Y4** | **Y5** | **Y6** | **Y7** | **Y8** | **Y9** | **Y10** | **Y11** | **Y12** | **Y13** | **Y14** | **Y15** | **Y16** | **Y17** | **Y18** | **Y19** | **Y20** | **Y21** | **Y22** |
| --- | --- | --- | --- | --- | --- | --- | --- | --- | --- | --- | --- | --- | --- | --- | --- | --- | --- | --- | --- | --- | --- | --- |
| S1 | 52.21 | 7.87 | 60.92 | -36.51 | -4.49 | -7.55 | -21.94 | -7.95 | 21.04 | 29.08 | 20.00 | 40.00 | 22.49 | 31.16 | 38.03 | 36.24 | 21.63 | 36.90 | 55.32 | -16.60 | 54.31 | -23.95 |
| S2 | 55.01 | 7.77 | 48.75 | -37.45 | -4.11 | -11.46 | -16.07 | -16.13 | 15.95 | 18.63 | 20.00 | 40.00 | 20.56 | 33.61 | 37.07 | 36.20 | 25.86 | 37.99 | 49.02 | -25.35 | 52.89 | -22.77 |
| S3 | 52.26 | 9.84 | 56.73 | -35.79 | -4.17 | -8.41 | -11.01 | -11.31 | 16.01 | 19.38 | 20.00 | 40.00 | 20.18 | 30.12 | 37.92 | 35.77 | 19.96 | 38.04 | 52.73 | -23.95 | 55.75 | -21.24 |
| S4 | 45.86 | 9.84 | 59.44 | -38.46 | -11.03 | 40.00 | -28.76 | -23.55 | 29.90 | 24.54 | 20.00 | -3.10 | 19.06 | 31.00 | 38.11 | 36.26 | 21.23 | 36.56 | 47.67 | -27.14 | 54.36 | -19.59 |
| S5 | 47.49 | 9.84 | 54.77 | -38.62 | -15.96 | -11.05 | -13.88 | -21.91 | 20.04 | 23.14 | 20.00 | -7.04 | 19.06 | 33.17 | 37.33 | 36.35 | 25.47 | 37.05 | 48.03 | -23.95 | 56.99 | -22.29 |
| S6 | 43.79 | 11.72 | 58.21 | -37.44 | -7.03 | -11.31 | -25.45 | -9.22 | 20.09 | 26.00 | 20.00 | -10.93 | 18.04 | 29.99 | 38.12 | 35.98 | 19.28 | 36.90 | 45.54 | -21.41 | 57.81 | -22.12 |
| S7 | 48.36 | 9.54 | 61.44 | -36.64 | -4.28 | -11.39 | -27.06 | -8.22 | 28.77 | 27.87 | 20.00 | -17.66 | 20.25 | 29.99 | 38.41 | 36.18 | 18.42 | 38.29 | 47.86 | -20.22 | 53.89 | -22.93 |
| S8 | 50.78 | 6.43 | 55.61 | -35.39 | -4.34 | -11.41 | -23.19 | -8.12 | 14.11 | 26.43 | 20.00 | -21.31 | 21.12 | 32.54 | 37.76 | 36.41 | 24.24 | 38.06 | 50.49 | -15.62 | 52.02 | -23.38 |
| S9 | 47.70 | 6.43 | 59.79 | -35.04 | -4.65 | -8.04 | -21.94 | -7.79 | 21.06 | 28.62 | 20.00 | -24.19 | 19.70 | 29.93 | 38.22 | 35.98 | 18.66 | 36.90 | 49.47 | -18.85 | 52.02 | -21.60 |
| S10 | 46.98 | 10.75 | 62.37 | -37.32 | -3.90 | -15.92 | -30.76 | -7.97 | 29.52 | 21.66 | 20.00 | 20.00 | 22.16 | 29.43 | 38.43 | 36.15 | 17.27 | 37.32 | 45.78 | -22.12 | 52.02 | -24.08 |
| S11 | 49.88 | 9.09 | 57.52 | -38.42 | -13.07 | -11.19 | -24.23 | -19.93 | 21.91 | 22.89 | 20.00 | 20.00 | 20.25 | 31.30 | 38.01 | 36.20 | 21.82 | 37.59 | 49.00 | -20.64 | 54.23 | -21.03 |
| S12 | 46.89 | 9.09 | 60.80 | -36.06 | -10.39 | -11.39 | -27.11 | -15.91 | 23.58 | 26.83 | 20.00 | 20.00 | 20.19 | 29.41 | 38.33 | 36.02 | 17.40 | 38.19 | 45.63 | -19.79 | 54.01 | -21.03 |
| S13 | 48.82 | 9.09 | 60.76 | -39.39 | -2.09 | -8.82 | -20.25 | -15.91 | 17.77 | 38.06 | -28.90 | -3.14 | -2.81 | 30.24 | 38.32 | 36.21 | 19.20 | 33.55 | 45.11 | -18.40 | 52.61 | -23.81 |
| S14 | 50.72 | 8.30 | 55.28 | -39.68 | -3.70 | -19.36 | -11.63 | -17.58 | 21.05 | 38.06 | -30.47 | -6.15 | -4.12 | 32.60 | 37.70 | 36.40 | 24.40 | 27.15 | 43.63 | -21.60 | 52.08 | -18.85 |
| S15 | 48.57 | 6.02 | 59.35 | -39.68 | -20.60 | -16.01 | -24.08 | -7.59 | 22.75 | 38.06 | -27.99 | -3.31 | -2.13 | 29.96 | 38.18 | 36.00 | 18.88 | 37.81 | 39.51 | -21.60 | 52.46 | -15.94 |

**Table 8.** Normalized S/N ratios at all runs.

| **Responses** | **Y1** | **Y2** | **Y3** | **Y4** | **Y5** | **Y6** | **Y7** | **Y8** | **Y9** | **Y10** | **Y11** | **Y12** | **Y13** | **Y14** | **Y15** | **Y16** | **Y17** | **Y18** | **Y19** | **Y20** | **Y21** | **Y22** |
| --- | --- | --- | --- | --- | --- | --- | --- | --- | --- | --- | --- | --- | --- | --- | --- | --- | --- | --- | --- | --- | --- | --- |
| S1 | 0.75 | 0.32 | 0.89 | 0.68 | 0.87 | 0.80 | 0.55 | 0.02 | 0.56 | 0.46 | 0.00 | 0.00 | 0.00 | 0.42 | 0.71 | 0.74 | 0.51 | 0.88 | 1.00 | 0.09 | 0.39 | 0.98 |
| S2 | 1.00 | 0.31 | 0.00 | 0.48 | 0.89 | 0.87 | 0.26 | 0.54 | 0.88 | 1.00 | 0.00 | 0.00 | 0.07 | 1.00 | 0.00 | 0.67 | 1.00 | 0.97 | 0.60 | 0.84 | 0.15 | 0.84 |
| S3 | 0.75 | 0.67 | 0.59 | 0.84 | 0.89 | 0.82 | 0.00 | 0.23 | 0.88 | 0.96 | 0.00 | 0.00 | 0.09 | 0.17 | 0.62 | 0.00 | 0.31 | 0.98 | 0.84 | 0.72 | 0.64 | 0.65 |
| S4 | 0.18 | 0.67 | 0.78 | 0.26 | 0.52 | 0.00 | 0.90 | 1.00 | 0.00 | 0.70 | 0.00 | 0.67 | 0.13 | 0.38 | 0.76 | 0.76 | 0.46 | 0.84 | 0.52 | 1.00 | 0.40 | 0.45 |
| S5 | 0.33 | 0.67 | 0.44 | 0.23 | 0.25 | 0.86 | 0.15 | 0.90 | 0.62 | 0.77 | 0.00 | 0.73 | 0.13 | 0.89 | 0.19 | 0.91 | 0.95 | 0.89 | 0.54 | 0.72 | 0.86 | 0.78 |
| S6 | 0.00 | 1.00 | 0.69 | 0.48 | 0.73 | 0.86 | 0.73 | 0.10 | 0.62 | 0.62 | 0.00 | 0.79 | 0.17 | 0.14 | 0.77 | 0.32 | 0.23 | 0.88 | 0.38 | 0.50 | 1.00 | 0.76 |
| S7 | 0.41 | 0.62 | 0.93 | 0.66 | 0.88 | 0.87 | 0.81 | 0.04 | 0.07 | 0.52 | 0.00 | 0.90 | 0.08 | 0.14 | 0.99 | 0.65 | 0.13 | 1.00 | 0.53 | 0.40 | 0.32 | 0.86 |
| S8 | 0.62 | 0.07 | 0.50 | 0.92 | 0.88 | 0.87 | 0.62 | 0.03 | 1.00 | 0.60 | 0.00 | 0.96 | 0.05 | 0.74 | 0.51 | 1.00 | 0.81 | 0.98 | 0.69 | 0.00 | 0.00 | 0.91 |
| S9 | 0.35 | 0.07 | 0.81 | 1.00 | 0.86 | 0.81 | 0.55 | 0.01 | 0.56 | 0.49 | 0.00 | 1.00 | 0.10 | 0.12 | 0.85 | 0.33 | 0.16 | 0.88 | 0.63 | 0.28 | 0.00 | 0.70 |
| S10 | 0.28 | 0.83 | 1.00 | 0.51 | 0.90 | 0.94 | 1.00 | 0.02 | 0.02 | 0.84 | 0.00 | 0.31 | 0.01 | 0.00 | 1.00 | 0.60 | 0.00 | 0.91 | 0.40 | 0.56 | 0.00 | 1.00 |
| S11 | 0.54 | 0.54 | 0.64 | 0.27 | 0.41 | 0.86 | 0.67 | 0.77 | 0.51 | 0.78 | 0.00 | 0.31 | 0.08 | 0.45 | 0.69 | 0.67 | 0.53 | 0.94 | 0.60 | 0.44 | 0.38 | 0.63 |
| S12 | 0.28 | 0.54 | 0.89 | 0.78 | 0.55 | 0.87 | 0.82 | 0.52 | 0.40 | 0.58 | 0.00 | 0.31 | 0.09 | 0.00 | 0.93 | 0.39 | 0.02 | 0.99 | 0.39 | 0.36 | 0.34 | 0.63 |
| S13 | 0.45 | 0.54 | 0.88 | 0.06 | 1.00 | 0.82 | 0.47 | 0.52 | 0.77 | 0.00 | 0.97 | 0.67 | 0.95 | 0.20 | 0.92 | 0.68 | 0.23 | 0.57 | 0.35 | 0.24 | 0.10 | 0.97 |
| S14 | 0.62 | 0.40 | 0.48 | 0.00 | 0.91 | 1.00 | 0.03 | 0.63 | 0.56 | 0.00 | 1.00 | 0.72 | 1.00 | 0.76 | 0.46 | 0.98 | 0.83 | 0.00 | 0.26 | 0.52 | 0.01 | 0.36 |
| S15 | 0.43 | 0.00 | 0.78 | 0.00 | 0.00 | 0.94 | 0.66 | 0.00 | 0.45 | 0.00 | 0.95 | 0.67 | 0.93 | 0.13 | 0.81 | 0.37 | 0.19 | 0.96 | 0.00 | 0.52 | 0.08 | 0.00 |

**Table 9.** Absolute Difference/ Quality Loss function at all levels.

| **Responses** | **∆_Y1_** | **∆_Y2_** | **∆_Y3_** | **∆_Y4_** | **∆_Y5_** | **∆_Y6_** | **∆_Y7_** | **∆_Y8_** | **∆_Y9_** | **∆_Y10_** | **∆_Y11_** | **∆_Y12_** | **∆_Y13_** | **∆_Y14_** | **∆_Y15_** | **∆_Y16_** | **∆_Y17_** | **∆_Y18_** | **∆_Y19_** | **∆_Y20_** | **∆_Y21_** | **∆_Y22_** |
| --- | --- | --- | --- | --- | --- | --- | --- | --- | --- | --- | --- | --- | --- | --- | --- | --- | --- | --- | --- | --- | --- | --- |
| S1 | 0.25 | 0.68 | 0.11 | 0.32 | 0.13 | 0.20 | 0.45 | 0.98 | 0.44 | 0.54 | 1.00 | 1.00 | 1.00 | 0.58 | 0.29 | 0.26 | 0.49 | 0.12 | 0.00 | 0.91 | 0.61 | 0.02 |
| S2 | 0.00 | 0.69 | 1.00 | 0.52 | 0.11 | 0.13 | 0.74 | 0.46 | 0.12 | 0.00 | 1.00 | 1.00 | 0.93 | 0.00 | 1.00 | 0.33 | 0.00 | 0.03 | 0.40 | 0.16 | 0.85 | 0.16 |
| S3 | 0.25 | 0.33 | 0.41 | 0.16 | 0.11 | 0.18 | 1.00 | 0.77 | 0.12 | 0.04 | 1.00 | 1.00 | 0.91 | 0.83 | 0.38 | 1.00 | 0.69 | 0.02 | 0.16 | 0.28 | 0.36 | 0.35 |
| S4 | 0.82 | 0.33 | 0.22 | 0.74 | 0.48 | 1.00 | 0.10 | 0.00 | 1.00 | 0.30 | 1.00 | 0.33 | 0.87 | 0.62 | 0.24 | 0.24 | 0.54 | 0.16 | 0.48 | 0.00 | 0.60 | 0.55 |
| S5 | 0.67 | 0.33 | 0.56 | 0.77 | 0.75 | 0.14 | 0.85 | 0.10 | 0.38 | 0.23 | 1.00 | 0.27 | 0.87 | 0.11 | 0.81 | 0.09 | 0.05 | 0.11 | 0.46 | 0.28 | 0.14 | 0.22 |
| S6 | 1.00 | 0.00 | 0.31 | 0.52 | 0.27 | 0.14 | 0.27 | 0.90 | 0.38 | 0.38 | 1.00 | 0.21 | 0.83 | 0.86 | 0.23 | 0.68 | 0.77 | 0.12 | 0.62 | 0.50 | 0.00 | 0.24 |
| S7 | 0.59 | 0.38 | 0.07 | 0.34 | 0.12 | 0.13 | 0.19 | 0.96 | 0.93 | 0.48 | 1.00 | 0.10 | 0.92 | 0.86 | 0.01 | 0.35 | 0.87 | 0.00 | 0.47 | 0.60 | 0.68 | 0.14 |
| S8 | 0.38 | 0.93 | 0.50 | 0.08 | 0.12 | 0.13 | 0.38 | 0.97 | 0.00 | 0.40 | 1.00 | 0.04 | 0.95 | 0.26 | 0.49 | 0.00 | 0.19 | 0.02 | 0.31 | 1.00 | 1.00 | 0.09 |
| S9 | 0.65 | 0.93 | 0.19 | 0.00 | 0.14 | 0.19 | 0.45 | 0.99 | 0.44 | 0.51 | 1.00 | 0.00 | 0.90 | 0.88 | 0.15 | 0.67 | 0.84 | 0.12 | 0.37 | 0.72 | 1.00 | 0.30 |
| S10 | 0.72 | 0.17 | 0.00 | 0.49 | 0.10 | 0.06 | 0.00 | 0.98 | 0.98 | 0.16 | 1.00 | 0.69 | 0.99 | 1.00 | 0.00 | 0.40 | 1.00 | 0.09 | 0.60 | 0.44 | 1.00 | 0.00 |
| S11 | 0.46 | 0.46 | 0.36 | 0.73 | 0.59 | 0.14 | 0.33 | 0.23 | 0.49 | 0.22 | 1.00 | 0.69 | 0.92 | 0.55 | 0.31 | 0.33 | 0.47 | 0.06 | 0.40 | 0.56 | 0.62 | 0.37 |
| S12 | 0.72 | 0.46 | 0.11 | 0.22 | 0.45 | 0.13 | 0.18 | 0.48 | 0.60 | 0.42 | 1.00 | 0.69 | 0.91 | 1.00 | 0.07 | 0.61 | 0.98 | 0.01 | 0.61 | 0.64 | 0.66 | 0.37 |
| S13 | 0.55 | 0.46 | 0.12 | 0.94 | 0.00 | 0.18 | 0.53 | 0.48 | 0.23 | 1.00 | 0.03 | 0.33 | 0.05 | 0.80 | 0.08 | 0.32 | 0.77 | 0.43 | 0.65 | 0.76 | 0.90 | 0.03 |
| S14 | 0.38 | 0.60 | 0.52 | 1.00 | 0.09 | 0.00 | 0.97 | 0.37 | 0.44 | 1.00 | 0.00 | 0.28 | 0.00 | 0.24 | 0.54 | 0.02 | 0.17 | 1.00 | 0.74 | 0.48 | 0.99 | 0.64 |
| S15 | 0.57 | 1.00 | 0.22 | 1.00 | 1.00 | 0.06 | 0.34 | 1.00 | 0.55 | 1.00 | 0.05 | 0.33 | 0.07 | 0.87 | 0.19 | 0.63 | 0.81 | 0.04 | 1.00 | 0.48 | 0.92 | 1.00 |

**Table 10.** Grey relational coefficient (GRC) at all runs.

| **GRC** | GC_Y1_ | GC_Y2_ | GC_Y3_ | GC_Y4_ | GC_Y5_ | GC_Y6_ | GC_Y7_ | GC_Y8_ | GC_Y9_ | GC_Y10_ | GC_Y11_ | GC_Y12_ | GC_Y13_ | GC_Y14_ | GC_Y15_ | GC_Y16_ | GC_Y17_ | GC_Y18_ | GC_Y19_ | GC_Y20_ | GC_Y21_ | GC_Y22_ |
| --- | --- | --- | --- | --- | --- | --- | --- | --- | --- | --- | --- | --- | --- | --- | --- | --- | --- | --- | --- | --- | --- | --- |
| **Sample code** |  |  |  |  |  |  |  |  |  |  |  |  |  |  |  |  |  |  |  |  |  |  |
| S1 | 0.80 | 0.60 | 0.90 | 0.76 | 0.89 | 0.83 | 0.69 | 0.51 | 0.69 | 0.65 | 0.50 | 0.50 | 0.50 | 0.63 | 0.77 | 0.79 | 0.67 | 0.89 | 1.00 | 0.52 | 0.62 | 0.98 |
| S2 | 1.00 | 0.59 | 0.50 | 0.66 | 0.90 | 0.88 | 0.57 | 0.68 | 0.90 | 1.00 | 0.50 | 0.50 | 0.52 | 1.00 | 0.50 | 0.75 | 1.00 | 0.97 | 0.72 | 0.87 | 0.54 | 0.86 |
| S3 | 0.80 | 0.75 | 0.71 | 0.86 | 0.90 | 0.84 | 0.50 | 0.57 | 0.89 | 0.96 | 0.50 | 0.50 | 0.52 | 0.55 | 0.73 | 0.50 | 0.59 | 0.98 | 0.86 | 0.78 | 0.74 | 0.74 |
| S4 | 0.55 | 0.75 | 0.82 | 0.58 | 0.67 | 0.50 | 0.91 | 1.00 | 0.50 | 0.77 | 0.50 | 0.75 | 0.53 | 0.62 | 0.81 | 0.81 | 0.65 | 0.87 | 0.67 | 1.00 | 0.63 | 0.64 |
| S5 | 0.60 | 0.75 | 0.64 | 0.56 | 0.57 | 0.88 | 0.54 | 0.91 | 0.73 | 0.81 | 0.50 | 0.79 | 0.53 | 0.90 | 0.55 | 0.91 | 0.96 | 0.90 | 0.68 | 0.78 | 0.88 | 0.82 |
| S6 | 0.50 | 1.00 | 0.77 | 0.66 | 0.79 | 0.88 | 0.79 | 0.53 | 0.73 | 0.72 | 0.50 | 0.83 | 0.55 | 0.54 | 0.82 | 0.60 | 0.57 | 0.89 | 0.62 | 0.67 | 1.00 | 0.81 |
| S7 | 0.63 | 0.72 | 0.94 | 0.74 | 0.89 | 0.88 | 0.84 | 0.51 | 0.52 | 0.68 | 0.50 | 0.91 | 0.52 | 0.54 | 0.99 | 0.74 | 0.54 | 1.00 | 0.68 | 0.62 | 0.60 | 0.88 |
| S8 | 0.73 | 0.52 | 0.67 | 0.93 | 0.89 | 0.88 | 0.72 | 0.51 | 1.00 | 0.71 | 0.50 | 0.96 | 0.51 | 0.80 | 0.67 | 1.00 | 0.84 | 0.98 | 0.77 | 0.50 | 0.50 | 0.92 |
| S9 | 0.61 | 0.52 | 0.84 | 1.00 | 0.88 | 0.84 | 0.69 | 0.50 | 0.69 | 0.66 | 0.50 | 1.00 | 0.53 | 0.53 | 0.87 | 0.60 | 0.54 | 0.89 | 0.73 | 0.58 | 0.50 | 0.77 |
| S10 | 0.58 | 0.85 | 1.00 | 0.67 | 0.91 | 0.95 | 1.00 | 0.51 | 0.51 | 0.87 | 0.50 | 0.59 | 0.50 | 0.50 | 1.00 | 0.71 | 0.50 | 0.92 | 0.62 | 0.70 | 0.50 | 1.00 |
| S11 | 0.69 | 0.68 | 0.74 | 0.58 | 0.63 | 0.88 | 0.75 | 0.81 | 0.67 | 0.82 | 0.50 | 0.59 | 0.52 | 0.65 | 0.76 | 0.75 | 0.68 | 0.94 | 0.71 | 0.64 | 0.62 | 0.73 |
| S12 | 0.58 | 0.68 | 0.90 | 0.82 | 0.69 | 0.88 | 0.84 | 0.68 | 0.63 | 0.70 | 0.50 | 0.59 | 0.52 | 0.50 | 0.93 | 0.62 | 0.50 | 0.99 | 0.62 | 0.61 | 0.60 | 0.73 |
| S13 | 0.64 | 0.68 | 0.89 | 0.52 | 1.00 | 0.85 | 0.65 | 0.68 | 0.81 | 0.50 | 0.97 | 0.75 | 0.95 | 0.55 | 0.92 | 0.76 | 0.56 | 0.70 | 0.61 | 0.57 | 0.53 | 0.97 |
| S14 | 0.72 | 0.63 | 0.66 | 0.50 | 0.92 | 1.00 | 0.51 | 0.73 | 0.69 | 0.50 | 1.00 | 0.78 | 1.00 | 0.81 | 0.65 | 0.98 | 0.86 | 0.50 | 0.57 | 0.68 | 0.50 | 0.61 |
| S15 | 0.64 | 0.50 | 0.82 | 0.50 | 0.50 | 0.95 | 0.75 | 0.50 | 0.65 | 0.50 | 0.95 | 0.75 | 0.93 | 0.53 | 0.84 | 0.61 | 0.55 | 0.96 | 0.50 | 0.68 | 0.52 | 0.50 |
